# Development and validation of a comprehensive food literacy questionnaire for adolescents integrating knowledge, skills, and environmental influences

**DOI:** 10.1186/s12889-026-26969-2

**Published:** 2026-03-13

**Authors:** Gertrude G. Zeinstra, Lenneke M. van Bussel, Femke A. Hoefnagels, Geertje van Wijk, Marieke C.E. Battjes-Fries

**Affiliations:** 1 Wageningen Social & Economic Research, Consumer Research group, Wageningen University & Research, Droevendaalsesteeg 4, Wageningen, 6708 PB The Netherlands; 2 Wageningen Food & Biobased Research, Food, Health & Consumer Research group, Wageningen University & Research, Bornse Weilanden 9, Wageningen, 6708 WG The Netherlands; 3 Department of Nutrition and Health, Louis Bolk Instituut, Kosterijland 3-5, Bunnik, 3981 AJ The Netherlands

**Keywords:** Adolescent, Literacy, Eating, Food, Questionnaire design, Validation study

## Abstract

**Background:**

Food literacy refers to the individual’s capabilities to make informed food choices. While there are several conceptual frameworks for defining food literacy, food literacy questionnaires specifically designed for adolescents are scarce. This study aimed to develop and validate a comprehensive food literacy questionnaire for adolescents.

**Methods:**

The study consisted of three stages. First, a concept questionnaire was developed based on literature, expert sessions, and existing food literacy questionnaires. Secondly, a validation study was conducted among 673 Dutch secondary school adolescents aged 12–18 years. The questionnaire was evaluated and optimized based on the facility index, factor analyses, Cronbach’s alpha, and process evaluation questions. Thirdly, the optimized food literacy questionnaire was completed by 540 Dutch adolescents (grade 1–3) for further evaluation and validation.

**Results:**

The concept questionnaire consisted of 63 items covering four main concepts: *Knowledge*, *Skills*, *Social environment*, and *Physical environment*. Based on the factor analyses results, the latter two concepts were reclassified into *Self-control* and *Handling socio-cultural influences*, which improved reliability. Based on the validation study results, ten knowledge questions (too easy or too difficult) and six statements were removed. This resulted in an optimized 47‑item questionnaire. The results from the third phase showed that this questionnaire was sufficiently reliable (Cronbach’s alpha between 0.72 and 0.83) and no longer contained knowledge questions that were too difficult or too easy.

**Conclusions:**

The questionnaire that was developed to assess adolescents’ food literacy contains a wide range of aspects related to being capable of making informed and conscious food choices. After testing in two validation studies, this questionnaire with 47 items was found to be sufficiently valid, reliable, and feasible to be administered in secondary schools. This food literacy questionnaire can be widely used in adolescent research and practice.

**Supplementary Information:**

The online version contains supplementary material available at 10.1186/s12889-026-26969-2.

## Background

Healthy eating is essential for optimal growth, development, and health of young people [1]. However, the prevalence of overweight among children and adolescents worldwide is 20% [2], and 13% among Dutch 4-17-year-olds [3]. On the other hand, underweight and thinness is also present, with estimates between 3.5% and 8.2% among European adolescents aged 11–14 years [4]. These numbers show that current diets of youngsters are neither healthy nor sustainable. To reduce diet-related chronic diseases, greenhouse gas emissions, and biodiversity loss, a transition is needed to more healthy and sustainable eating patterns [5, 6].

Food preferences and dietary habits acquired in childhood and adolescence tend to persist into adulthood [7–10]. Therefore, children and adolescents are an important group to target for the development of healthy and sustainable eating patterns. Adolescence is a unique life stage, that is characterized by rapid and profound changes in cognitive, social, physical, and biological development [11]. Adolescence is also a critical transition period when looking at nutrition behaviour. Key drivers that particularly influence adolescents’ food choices cover different levels of the socio-ecological model [11–13] and encompass a greater autonomy, more purchasing power, a higher importance of the social environment (peers, social media), while there is also a strong desire for uniqueness and identity formation [11, 13–15]. At the same time, parents and the home environment still play an important role. A cross-sectional study in the Netherlands showed that food intake of the mother was stronger related to adolescents’ food intake than their best friends, and this was true for both healthy and unhealthy foods [16]. Furthermore, when considering food consumption at school, Dutch adolescents bring most of the food they consume from home [17, 18]. From a cognitive developmental point of view, adolescents enter the formal operational stage, which is characterized by the emergence of more abstract, systematic, and reflective thinking and the ability to consider hypothetical situations [19, 20].

To successfully make healthy and sustainable food choices, adolescents should be well-equipped to make informed choices while navigating through different environments. This capacity of an individual to plan, manage, select, prepare, and eat food to fulfil needs and determine food intake [21] is also called food literacy. There are various conceptual frameworks for defining food literacy, and there is no consensus among these frameworks [22]. A commonly used framework includes knowledge and competencies, thereby making a distinction between functional, relational, and critical competencies [19, 23–29]. In these frameworks, knowledge refers for example to knowledge about nutrients, portion size, food safety, product knowledge, and health. In some of these frameworks, knowledge is seen as a separate component, whereas in others, it is part of the functional competencies [19, 27, 29]. The different competencies refer to cooking and food shopping skills, discussing about food, executing food activities together, as well as being able to handle media and food label information, food budgeting, or understanding the broader food system [19, 27, 29]. There are also frameworks using the food chain as starting point. Here, production, distribution, selection, preparation & cooking, and intake are important domains for food literacy [30]. In general, most frameworks include a cognitive component (knowledge) and a skill component (competencies), but they differ in whether a behavioural component (such as food choices or healthy eating) is included or not. Furthermore, several broadly used food literacy frameworks do not acknowledge life-stage specific criteria [19, 31], whereas this seems an important aspect to consider to ensure that the concepts and questions match with the target group and their world of living.

Increasing the level of food literacy among youth is an important aim of the Dutch governmental program Jong Leren Eten (Learning to eat at a young age: https://www.jonglereneten.nl/). This program was initiated in 2017 by the Ministry of Agriculture, Fisheries, Food Security, and Nature. The program focuses on nutrition and operates at the interface of health and environmental education. The program aims to improve food literacy among children and young people (0–24 years) via educational and nutritional interventions to enable youngsters to make healthy and sustainable choices now and later in life. To assess the impact of the program activities, it would be valuable to have a tool that measures effects on food literacy accurately, standardly, and age-specific. This tool should assess the individual’s ability to make both healthy and sustainable food choices. Recent studies confirm this need for a valid and reliable tool to measure food literacy among adolescents [19, 32]. The need for a broad tool assessing multiple aspects of food literacy is also acknowledged [19, 32, 33]. Furthermore, there are still food literacy scales used with poor or questionable internal consistency [31].

A validated food literacy scale for Dutch adults (18+) is available and has a strong focus on healthy eating [34]. Recently, a food literacy questionnaire has been developed for Dutch primary-school-aged children, targeting 9–12-year-olds [35]. This questionnaire covers knowledge about both healthy and sustainable food aspects. However, there is no food literacy questionnaire for Dutch adolescents, attending secondary school (aged 12–18 years). Questionnaires for measuring food literacy among adolescents are scarce in general. Park et al. [36] developed a food literacy scale for Korean youth aged 8–18 years which was adapted from an adult version [30]. This 19-item scale covers the concepts production, distribution, selection, preparation & cooking, and intake which are scored on a 5-point Likert scale. In addition, a Danish food literacy scale was developed for adolescents 12–14 years [37]. This instrument covers five dimensions: to know, to do, to sense, to care, and to want. The 37-item questionnaire consists of 35 Likert-type items and two true/false items. The targeted age range of these instruments is broader [30] or narrower [37] than Dutch secondary school adolescents. Although adaptation studies could be useful, we aimed to develop an instrument that is unique to the Dutch context, because food literacy is highly contextual, influenced by the geography, its food system and the social and cultural context [19, 31].

The study aimed to develop and validate a comprehensive food literacy questionnaire for Dutch adolescents aged 12–18 years that covers both healthy and sustainability food aspects and matches their life period. This study fills an important gap in the existing literature by developing an instrument tailored to this particular age group.

### Methods

A three-stage approach with six underlying steps was used to develop and validate a food literacy questionnaire for adolescents. The development was guided by four criteria. These were derived from contextual requirements for the questionnaire’s application in the Netherlands and from the literature (i.e. [19, 31]). The questionnaire should:

1. encompass a broad range of food literacy aspects (covering both healthy and sustainable food aspects)
2. be able to assess differences between groups of adolescents
3. apply to the various grades and levels of secondary school
4. match the life stage and cognitive capacities of secondary school pupils (12-18y)

In the first stage, important concepts and items were generated based on literature and expert sessions. In the second stage, a cross-sectional survey was executed among adolescents from different grades and school levels. The questionnaire was evaluated based on the facility index, Factor Analysis, Cronbach’s alpha, and process evaluation questions. In the third phase, the adapted food literacy questionnaire was completed by adolescents of the first three grades of secondary schools. Further details are described in the following sections. The study protocol was approved by the Research Ethics Committee of Wageningen University & Research (Number 2023-043; Approval date: 6-10-2023).

### Stage 1: Development

#### Step 1: Identification of main concepts and items

First, existing concepts related to food and nutrition literacy were explored due to the lack of conceptual clarity in the field [22]. Based on current food literacy frameworks [21, 29, 37], an overarching framework was developed with initial main concepts that were relevant for adolescents’ food literacy. These main concepts were categorized according to the COM-B model, developed by Michie et al. [38], that identifies motivation, capability, and opportunity as the three key interacting factors for predicting behaviour.

Subsequently, an expert session was organized on a central location with a diverse group of public health professionals, such as nutritionists and health-promotion advisors (*N* = 8), teachers (*N* = 5), and academics (*N* = 9) working in the field of health promotion, food literacy, and adolescents. Twenty-two persons were invited; eight could attend the session in person. The aim of this 2-hour session was to identify main and sub-concepts, that were relevant for adolescents, grounded in theory, and combined with experiences from practice. The initial overarching framework with main concepts – combined with the different food choice stages: plan/ manage, select, prepare, eat [21, 36] – was presented to probe broad thinking. Via a brainstorm carousel, experts wrote down all aspects that they thought were relevant for adolescents’ food literacy. Moreover, during a plenary discussion, input was obtained on a suitable number of items (length of the questionnaire), potential answer categories, relevant design aspects for adolescents, and concepts that should not necessarily be included in the food literacy questionnaire. As a token for their contribution, the experts received a voucher.

To ensure that the chosen main concepts were meaningful and relevant for the target group, a 1-hour session was organized with four adolescents. We deliberately recruited pupils from the lowest two grades (age 12–14 years) to ensure that the perspective of the youngest adolescents was included. Comparable to the expert session, the adolescents were asked to think about all aspects that they thought were relevant for adolescents’ food literacy. This was done in an age-appropriate way with six topics for probing: planning, buying, preparing, eating, storing of food, and food production. Furthermore, the layout of the questionnaire was discussed (number of items, appropriate answer categories, and design aspects). The adolescents received a voucher to thank them for their participation.

#### Step 2: Development of long-list food literacy questionnaire

Based on the results from step 1, main key concepts (*Knowledge*,* Skills*, and *Social* and *Physical environment*) and sub-concepts for the adolescents’ food literacy questionnaire were determined. Existing food literacy questionnaires [30, 34, 37, 39, 40] and conceptual food literacy papers [23, 28, 29, 31, 41] were scanned for suitable items for the chosen (sub-)concepts. These items were adapted where needed and supplemented by the research team, taking the Dutch educational guidelines with key learning goals for nutrition into account [42]. The result of this step was a long-list of 131 questions for the food literacy questionnaire, including multiple-choice answer options for the knowledge questions.

#### Step 3: Content validation – evaluation of the long-list food literacy questionnaire

To ensure content validity, the same group of 22 health professionals, teachers, and academics as in step 1 was invited for a digital consultation round. They received the long-list questionnaire via email. They were asked to score the relevance of each item on a 5-point scale (1 = not at all relevant; 5 = highly relevant) [13, 34], to check clearness of the items & answer options, to indicate whether topics were missing or whether items were double, and to provide any suggestions for improvement (content-wise, wording, use of language, layout). Eleven experts provided feedback; they received a voucher as a token of appreciation.

**Fig. 1:**
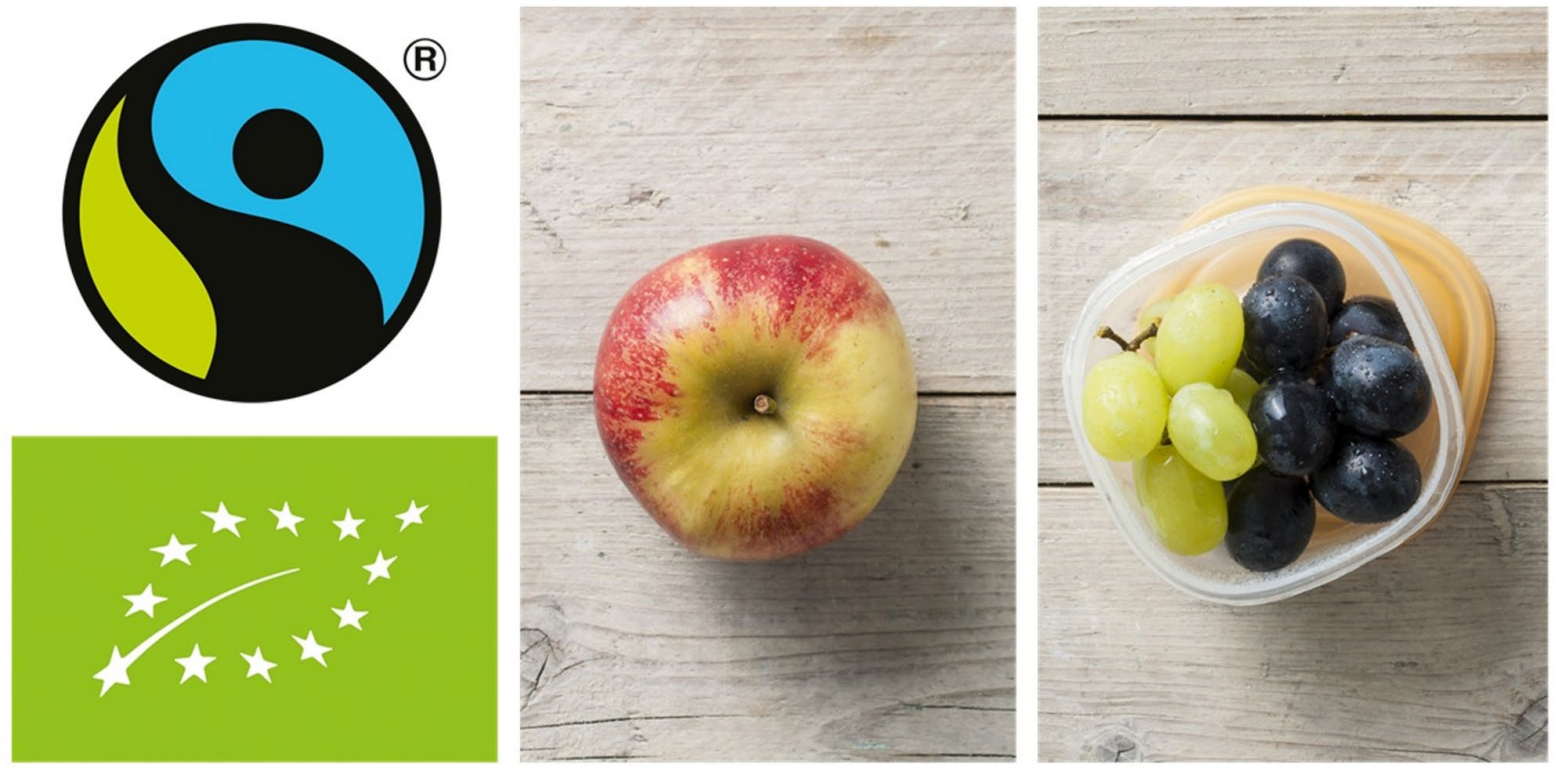
Pictures in the food literacy questionnaire that were used for clarification purpose: two logos and two images explaining one fruit portion (photo’s ©Voedingscentrum)

Based on the scores and feedback of the experts and extensive discussions within the research team, items were deleted, added, and revised. The researchers ensured that the questionnaire still covered different aspects of knowledge, skills, and the environment and that items were asked in a logical order. Care was taken that easy language was used: text was phrased as much as possible at B1 level, which is generally understood by the vast majority of the Dutch population [43]. Moreover, objective words such as adolescents and pupils were used instead of boys and girls to be gender-inclusive. This 63-item food literacy questionnaire version was programmed in Qualtrics survey software (Qualtrics, version 2023, Provo, UT, www.qualtrics.com). A few pictures were included for clarification purposes. These images illustrated key topics such as portion sizes and logos (see Fig. 1), ensuring that all respondents - regardless of their literacy level - could interpret the questions correctly. Permission to use these pictures was obtained from the Netherlands Nutrition Centre (Voedingscentrum). 

To support face validity, the programmed questionnaire was pilot tested among a small convenience sample of adolescents (*N* = 2). The pilot took about 45 min, and the adolescents received a voucher to thank them for their input. Three experts – from the same group that evaluated the long-list – also examined the updated questionnaire in Qualtrics. A few minor amendments were made to improve comprehensibility and readability of the food literacy questionnaire, that was subsequently used in the validation study.

### Stage 2: Scale testing

#### Step 4: Validation study among adolescents

Between October and December 2023, secondary schools and/ or teachers were recruited via convenience sampling. Different channels were used to reach a diverse group of adolescents concerning grade, education level, and region. Secondary schools that offered different levels of education (prevocational, senior general, and pre-university education) could participate, whereas secondary schools that provided special needs education were excluded. The research team phoned existing contacts, used social media, and asked partners who have contact with secondary schools to distribute the recruitment materials. An information leaflet explained the background, research aim, and the study procedures. The research team contacted 17 schools directly and 12 other schools showed their interest via an online form. Ten schools subscribed for the study, of which nine schools participated in the study.

After agreement of the school, a date was set with the teacher. Parents were informed about the study and could object to participation of their child if they wished so. All adolescents of a participating class were invited to join. We strived to include at least 630 secondary school pupils, in line with the guideline of at least 10 participants per item in a scale [44]. Inclusion criteria for the adolescents were: being able to read Dutch, willingness to participate, and no parental objection to participation.

Teachers received information and step-by-step instructions per email as well as the digital link to the questionnaire. Adolescents provided consent on the first page and completed the online questionnaire independently under supervision of their teacher. Pupils with parental objection or who did not want to participate in the study were given a task by their teacher. All teachers received a voucher per participating class in return for their help.

The questionnaire that the adolescents completed consisted of 81 items, with the 63 food literacy items being the first and main part. Furthermore, they completed ten social-demographic questions such as age, gender, educational level, school grade, food allergies in the family, habitual food buying and cooking frequency. These questions were at the end of the questionnaire. Eight process evaluation questions were included to examine adolescents’ experiences on completing the food literacy questionnaire. These questions were answered on a 3-point or 5-point scale and included the following topics: length and difficulty of the questionnaire, applicability for adolescents 12–18 years, and the use of pictures.

The recruitment procedure resulted in 34 school classes of nine different schools participating in the study. Of the 739 pupils opening the questionnaire, 673 agreed to participate (91%) and 66 pupils (9%) did not want to participate. The whole questionnaire (81 items) was completed by 569 pupils (85%).

#### Step 5: Data analyses, item reduction analysis, and optimization of questionnaire

Data-analyses were conducted in SPSS (IBM SPSS Statistics, version 28, IBM Corp., Armonk, N.Y., USA). Participants were included in the data analyses for a certain concept if they completed all questions from that concept. The following principles guided the data analyses. Firstly, the questionnaire should cover as many concepts as possible with as less questions as possible. Secondly, questions with hardly any variation or a ceiling effect (no room for improvement) should be omitted. Thirdly, questions that were not understood or too difficult should be excluded.

##### Food literacy knowledge questions

The answers to the knowledge questions were scored as correct or incorrect. Subsequently, the number of correct answers was calculated for the main concept of knowledge as well as for the sub-concepts. Average sum scores were calculated for the whole sample. Subsequently, the facility index was inspected. Usually, a cut-off of < 20% correct and > 80% correct is used [45]. With the development criteria for the questionnaire in mind and because the participants in the validation study were relatively highly educated (41% high; 49% middle; see Table 2), we heightened the upper limit to 85%. Thus, questions with less than 20% of the sample answering correct were regarded as too difficult. Questions with more than 85% of the sample answering correct were considered too easy.

##### Food literacy questions with 5-point answering scale

Means and standard deviations (SD) were calculated for the items in the questionnaire on the other concepts (*Skills*, *Social* and *Physical environment*). Questions with a mean score > 4.5 were considered unsuitable, since we assumed that with such a high score there was too little room for improvement. A Principal Axis Factoring was performed to explore the underlying structure of the items and to explore whether items could be left out (not loading on a main factor). Since the food literacy concepts may be correlated, an Oblimin rotation was applied. Besides, Cronbach’s alpha was calculated for the items within a main and sub-concept to determine the internal consistency. A Cronbach’s alpha of above 0.80 was defined as good reliability, whereas a value of 0.70 was considered acceptable [46, 47]. A Cronbach’s alpha below 0.5 was considered unacceptable. The average score with SD was calculated for each main and sub-concept.

##### Demographic and process variables

Frequencies (for categorical variables) or means with SD were calculated for the socio-demographic and process evaluation questions.

### Stage 3: Testing optimized questionnaire

#### Step 6: Second validation study among adolescents

With the optimized food literacy questionnaire (47 items), a second study was conducted among a different sample of secondary school pupils. For the recruitment (March & April 2024), health professionals were asked whether they had connections with secondary schools. The research team contacted those schools via similar procedures as in the validation study (step 4). We again strived to include adolescents from different levels of education, but now focused on younger adolescents (grade 1–3 of Dutch secondary schools) since this group was less represented in the first study. Again, all adolescents were invited to participate. Inclusion criteria were: being able to read Dutch, willing to participate (giving consent on the first page of the online questionnaire), and no parental objection to participation. Adolescents completed the online questionnaire in class under supervision of their teacher. Teachers received a voucher per class to thank them for their participation.

The research team invited 105 schools, of which 30 schools responded. Eleven schools subscribed for the study, of which eight participated in the study with a total of twenty school classes. Of the 579 pupils opening the survey, 540 (93%) agreed to participate and 39 pupils (7%) did not want to participate. The whole questionnaire was completed by 474 pupils (88%).

Similarly, as in step 5, the number of correct answers for the knowledge questions were calculated to inspect the data. For the other questions, the means and SD were calculated. The facility index and Cronbach’s alpha were used to evaluate the optimized questionnaire. Finally, differences in food literacy scores (main concepts) between groups were calculated for grade and educational level (one-way ANOVA with Tukey HSD post-hoc test), and for gender (independent samples t-test), to explore whether the questionnaire was sensitive enough to pick up differences between groups.

## Results

### Stage 1: Development of the questionnaire

Step 1 led to four main concepts that encompass adolescents’ food literacy: *Knowledge*, *Skills*, the *Social environment*, and the *Physical environment*, in line with the COM-B model that was used to structure the concepts. Motivation was not included, because food literacy was defined as being capable of making informed food choices independent of the current motivation at that moment or in that setting. Similarly, eating behaviour was also not included, as this refers to a behavioural outcome, which goes beyond the determinant of being capable of making informed food choices. The developed food literacy long-list consisted of 131 items. The main concepts and sub-concepts with the corresponding number of items are shown in Table 1.

**Table 1:** Identified main and sub-concepts, with number of items, to assess adolescents’ food literacy

Main concept	Sub-concepts	Number of items
Knowledge	1) Knowledge about healthy eating	18
	2) Knowledge on link between nutrition and health	4
	3) Knowledge about foods	19
Skills	1) Planning skills	19
	2) Selection skills	13
	3) Preparation skills	17
	4) Eating skills	4
	5) Storage skills	8
Social environment	1) Social influences	11
	2) Eating pleasure & proper relationship with food	10
Physical environment	1) Influence of physical food environment	8
	2) Cultural context	
Total		131

Based on the digital consultation round (content validation), 62 items were removed. These were considered not or less relevant (mean score < 4) or confusing for adolescents according to the experts. Ten items were added to mitigate double‑barrelled questions and to include topics experts indicated were missing. Finally, another 16 items were removed from the questionnaire since these items duplicated another item or the formulation of the item remained unclear. The result of this process was a set of 63 items: 24 multiple choice questions (to capture *Knowledge*) and 39 statements on a 5-point scale (capturing *Skills*, the *Social* and *Physical environment*). See Appendix 1 for all 63 items of the food literacy scale.

### Stage 2: Results of the validation study among secondary school pupils

The nine schools covered mainly the middle and northern part of the Netherlands. The adolescents’ mean age was 14.9 ± 1.6 years old, with slightly more girls (52%) than boys (41%; see Table 2). Most of them attended a middle (49%) or high educational level (41%). Most participants were in the 4th grade (32%), with about 20% of the sample in the 2nd, 3rd, and 5th grades. 30% indicated to have food allergies within the family. Almost 40% of the adolescents prepared a dish at home a few times per week, whereas 35% reported to do this 1–2 days per month or less, and 14% to do this never. About a quarter reported to buy their lunch at or around school 1–2 times per week (24%), whereas the majority did this only 1–2 days per month (29%), a few times per year (26%) or never (18%).

**Table 2:** Socio-demographic and food-related characteristics of the adolescents participating in the validation study (*N* = 573)

		*N*	%
Age (in years, mean ± SD, range)		14,9 ± 1.6	[10–19 years]
Gender	*Boy*	234	41
	*Girl*	300	52
	*Other/ don’t want to say*	39	7
Educational level (*N* = 541)	*Low (Prevocational secondary education)*	45	9
	*Middle (Senior general secondary education)*	267	49
	*High (Pre-university education)*	222	41
	*Other*	7	1
Grade	*1*	25	4
	*2*	100	18
	*3*	119	21
	*4*	182	32
	*5*	108	19
	*6*	39	7
Food allergies (*N* = 573)	*Yes*	170	30
	*No*	337	59
	*Don’t know*	66	12
Preparing a dish at home (*N* = 570)	*6–7 days per week*	29	5
	*4–5 days per week*	43	8
	*1–3 days per week*	220	39
	*1–2 days per month or less*	197	35
	*Never*	81	14
Buying lunch - at school or in a shop close to school (*N* = 569)	*4–5 days per week*	23	4
	*1–3 days per week*	136	24
	*1–2 days per month*	165	29
	*Few times per year*	145	26
	*Never*	100	18

Completion of the whole questionnaire took on average 16.1 ± 10.2 min. In general, the adolescents were satisfied with the questionnaire, with more than 70% of the adolescents providing positive answers for the various process evaluation aspects. Almost half of them (48%) indicated that the questionnaire was too long, pointing to the need to shorten the questionnaire.

The average number of correct responses for the *Knowledge* items ranged from 6% to 96%. Eight items were answered correctly by more than 85% of the pupils. These items were considered too easy and omitted from the questionnaire. One question (“What disease could you get from this?”) was omitted based on teacher feedback (difficult question, but answers were recited in class). Furthermore, there was one question that was answered correctly by less than 20% of the sample (“Which product contains the most vitamin C?”), this question was regarded as too difficult. As a result, the sub-concept *Knowledge on link between nutrition and health* was excluded from the questionnaire, whereas the sub-concepts *Knowledge about healthy eating* and *Knowledge about foods* were maintained with seven items each. For the statements covering *Skills*, the *Social* and *Physical environment*, four statements had a score above 4.5 (all within the sub-concept of preparation), and one item within the sub-concept planning scored ~ 4.5. These items were therefore omitted from the questionnaire. The average scores and SD per item and the (sub-)concepts are shown in Appendix 1, including the items that were omitted in this step.

The factor analysis resulted in eight factors, together explaining 43% of the variance. The results confirmed the underlying structure for *Skills*: planning, selection, preparation, and storage, except for one storage item, which was omitted to accommodate the need for brevity. The factor analysis showed a different underlying structure for the *Social* and *Physical environment* items. Since the internal consistency of the original *Social* (CA = 0.654; with 0.551 and 0.443 for sub-concepts) and *Physical environment* concepts (CA = 0.581) was insufficient, and the two new underlying concepts were interpretable, these new concepts were agreed on and used in further steps. The new concept *Self-control* and the original *Skills* sub-concepts had satisfactory Cronbach’s alpha scores (see Table 3), with *Handling socio-cultural influences* very close to acceptable (CA = 0.675). Deleting one or more items did not improve internal consistency. The validation process resulted in an updated questionnaire with 47 questions (14 *Knowledge* items and 33 statements on the three other concepts).

**Table 3:** Internal consistency for the (sub-)concepts of the food literacy questionnaire in the validation study

(Sub)concept	Number of items	Cronbach’s alpha
Skills	27	0.916
- Planning	7	0.770
- Selection	6	0.755
- Preparation	9	0.829
- Storage	5	0.748
Self-control	6	0.698
Handling socio-cultural influences	6	0.675

### Stage 3: Testing optimized questionnaire

Most participating schools were in the northern part of the Netherlands. Participants’ mean age was 13.2 ± 1.1 years, with slightly more boys (48%) than girls (42%; see Table 4). Almost half of them (46%) attended lower education, and a third (35%) middle education. About half of the students (48%) were in the 1^st^ grade, with 39% in the 2^nd^ grade and 14% in the 3^rd^ grade. A quarter (24%) reported to have food allergies within their family. Preparing a dish at home was mostly done 1–3 days per week (31%) or 1–2 days per month or less (40%). 30% of the adolescents bought lunch at or nearby school 1–3 days per week, whereas most of them did this less often (48%) or never (18%). Completion time of the full questionnaire was 17 ± 9.6 min.

**Table 4:** Socio-demographic and food-related characteristics of the adolescents participating in the optimization study (*N* = 474)

		*N*	%
Age (in years, mean ± SD, range)		13.2 ± 1.1	[10–16 years]
Gender	*Boy*	226	48
	*Girl*	201	42
	*Other or didn’t want to say*	47	10
Educational level (*N* = 466)	*Low (Pre vocational secondary education)*	214	46
	*Middle (Senior general secondary education)*	162	35
	*High (Pre-university education)*	90	19
Grade	*1*	227	48
	*2*	183	39
	*3*	64	14
Food allergies (*N* = 474)	*Yes*	124	26
	*No*	239	50
	*Don’t know*	111	23
Preparing a dish at home (*N* = 474)	*6–7 days per week*	21	4
	*4–5 days per week*	36	8
	*1–3 days per week*	145	31
	*1–2 days per month or less*	189	40
	*Never*	83	18
Buying lunch - at school or in a shop close to school (*N* = 474)	*4–5 days per week*	42	9
	*1–3 days per week*	143	30
	*1–2 days per month*	126	27
	*Few times per year*	100	21
	*Never*	63	13

The participants answered about half of the *Knowledge* questions correctly with an average score of 6.9 ± 2.4 out of 14 (See Table 5). The average score for the sub-concepts was 3.7 ± 1.5 out of seven for *Knowledge about healthy eating* and 3.2 ± 1.5 out of seven for *Knowledge about foods*. The percentage of correct answers per question ranged between 26% and 80%, indicating that there were no questions which were too difficult (correct by less than 20%) or too easy (correct by more than 85%). The overall knowledge score was higher for adolescents who followed pre-university education (8.8) than for adolescents who followed pre-vocational secondary education (6.1; *p* < 0.001) or senior general secondary education (6.9; *p* < 0.001). Moreover, senior general secondary education adolescents scored higher on knowledge than pre-vocational secondary education adolescents (*p* = 0.003). A similar pattern with significant differences was seen for the two knowledge sub-concepts separately (*p*-values < 0.018). Third grade pupils also scored higher (8.6) on the main concept of *Knowledge* than 1^st^ grade (6.3; *p* < 0.001) or 2^nd^ (7.0; *p* < 0.001) grade pupils, which was also true for the two sub-concepts (*p*-values < 0.013). Girls had a higher *Knowledge* score than boys (*p* = 0.01). This difference was also present for the sub-concept *Knowledge about foods* (*p* = 0.006), but not for the sub-concept *Knowledge about healthy eating* (*p* = 0.15).

**Table 5:** Internal consistency, means, and standard deviations for the updated (sub-)concepts of the food literacy questionnaire

	*N*	Mean	SD	Cronbach’s alpha	Number of items
Knowledge *	487	6.9	2.4	*N*.A.	14
- Knowledge about healthy eating	487	3.7	1.5	N.A.	7
- Knowledge about foods	487	3.2	1.5	N.A.	7
Skills **					
- Planning	481	3.23	0.84	0.804	7
- Selection	477	3.57	0.89	0.834	5
- Preparation	476	3.71	0.94	0.764	5
- Storage	475	3.53	0.97	0.803	4
Self-control	506	3.63	0.73	0.731	6
Handling socio-cultural influences	502	3.25	0.81	0.715	6

* Number of correct answers

** On a 5-point scale

**Fig. 2:**
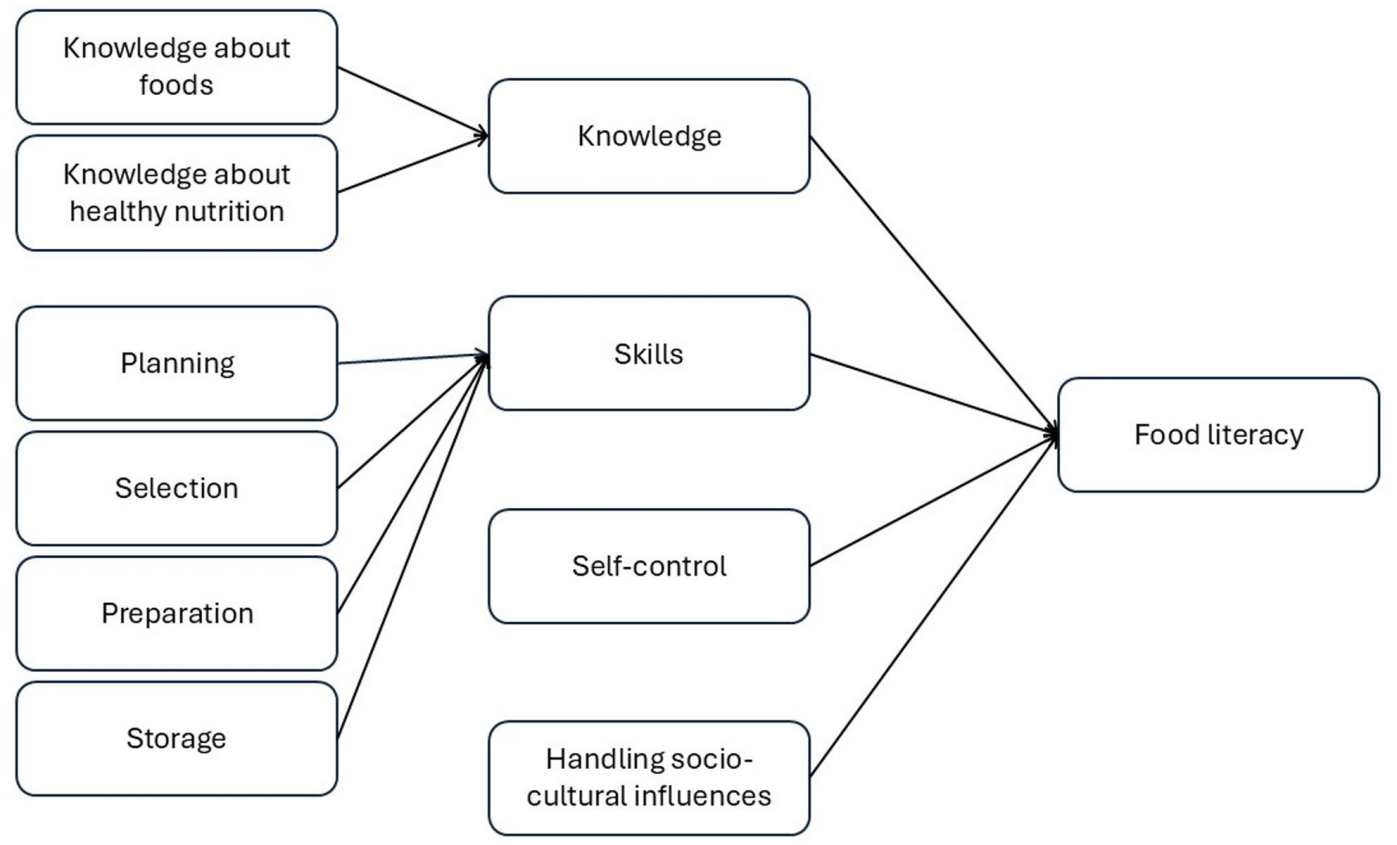
Conceptual model of the food literacy questionnaire for adolescents

Table 5 also shows the average scores for the statements belonging to the main concepts *Skills*, *Self*-*control* and *Handling socio-cultural influences*. The adolescents scored on average neutral to positive on these statements, with scores between three and four on a 5-point scale. They scored highest on *Preparation skills* and lowest on *Planning skills*. All (sub-)concepts had a Cronbach’s alpha > 0.7, indicating acceptable to good internal consistency. The conceptual model of the food literacy questionnaire is shown in Fig. 2.

There were significant differences between education levels in mean scores for *Planning skills* (*p* = 0.005), *Preparation skills* (*p* = 0.002), *Storage skills* (*p* = 0.006), and *Handling socio-cultural influences* (*p* < 0.001). Adolescents who followed pre-university education had higher scores than adolescents who followed senior general secondary education (all *p* < 0.05; except *Planning skills*: *p* = 0.05). Moreover, pre-university education adolescents scored significantly higher than pre-vocational secondary education adolescents on these four concepts (all *p* ≤ 0.005). Third grade pupils scored higher on *Storage skills* (*p* = 0.02) and *Handling socio-cultural influences* (*p* = 0.03) than 1^st^ grade pupils. Girls had higher *Preparation skills* scores than boys (*p* < 0.001), however scored lower on *Handling socio-cultural influences* than boys (*p* = 0.04).

## Discussion

This study aimed to develop and validate a comprehensive food literacy questionnaire specifically for adolescents. Because there was no tool available that measures food literacy accurately, standardly and age-specific among adolescents 12–18 years old, the new developed questionnaire fulfils a need. The developed questionnaire showed satisfactory internal consistency, validity, and discriminative validity. The final 47-item instrument is comprehensive including both healthy and sustainable aspects and covering the concepts of knowledge, skills, and the ability to deal with influences from the environment. The results showed that the questionnaire is applicable in various ages (12–18 years), different school levels, and easy to complete.

The current questionnaire is specifically focused on adolescents 12–18 years to fill a gap: previous food literacy questionnaires did not cover this age range [30, 37]. Earlier research pointed out that certain food literacy concepts develop at different ages, i.e. functional food-related competencies develop later than relational competencies [19, 29]. This, together with the fact that adolescence encompasses a specific life stage regarding cognitive developmental skills and their life context (i.e. greater autonomy, purchasing power, social media use, and the importance of identity formation), highlights the importance of developing an age-appropriate tool that captures the unique life stage of adolescence.

In line with various food literacy scales and conceptual frameworks [19, 25, 29], our food literacy scale includes the main concept of *Knowledge* and *Skills*, the more individual determinants of food choice in both the COM-B model and the socio-ecological model [48, 49]. The concepts of *Self-Control* and *Handling socio-cultural influences* cover the larger social and physical environment of these models, including the family rules, cultural norms, and media influence. Although Park et al. [30] also included items about the food system, previously developed food literacy scales had less focus on this broader food system [23, 37, 39]. Facing the current planetary health challenges [5, 6], it is important that food literacy pays attention to both healthy and sustainable dietary behaviours.

There are parallel concepts in our questionnaire and the Danish [37] and Korean one [30], but also differences. Our questionnaire pays more attention to storage skills, self-control (feeling full, balancing food intake, and dealing with temptations in the environment), as well as the influence of social media (i.e. influencers). These concepts are not included in the Danish and Korean ones. Furthermore, our concept of food preparation includes more technical skills than the one of Park et al. [30]. On the other hand, their food literacy scale includes several items that are more related to behaviour, such as “I usually check for the food’s country of origin (production)”; “I usually willingly participate in preparing or cooking meals (preparation)”, and “I usually try to eat a variety of food groups, including grains, fish, meat, vegetables, fruits, dairy (intake)”. In contrast, we deliberately chose not to include behaviour in our food literacy questionnaire, since we aimed to focus on adolescents’ knowledge and their capacities to do as well as to cope with environmental influences. This operationalization of food literacy comes very close to the definition of Ares et al. [19], who define food literacy as the abilities, knowledge, and skills that are needed to interact within the components of the food system in a way that children and adolescents can develop healthy and sustainable eating habits throughout the lifespan.

Another advantage of leaving out food choice behaviour, is that our questionnaire moves away from providing implicit norms about healthy and sustainable food choices, and the risk of providing implicit signals such as ‘you don’t eat healthy enough’. Adolescents are in a vulnerable phase for the onset of eating disorders [50, 51], so leaving out such signals can be beneficial. It also fits well in the educational setting, where the focus is on competence learning. We think it may be easier for teachers to explore what students already know and have learned, rather than engaging in potentially sensitive discussions about good and bad food choices. Moreover, adolescence is a typical life stage where youngsters develop more autonomy, they form their identity, they experiment and oppose to their parents [15, 52]. This means that adolescents will not always make healthy and sustainable food choices, even though they would have the capabilities to do so. This does not mean that we should neglect their actual food choices, or refrain from trying to improve them, but for assessing their food literacy, we aimed to focus on their capabilities.

Conceptual papers show lots of concepts that are part of being food literate [19, 27–29, 53]. However, it is challenging to cover all these aspects in a measurement tool that is still feasible to complete within a reasonable time frame. As a consequence, there is a high variation in the number of items in existing food literacy questionnaires, varying from 5 to 61 [31]. Existing international food literacy questionnaires, that focus on adolescents, consist of 19 [30] or 37 items [37]. The Dutch food literacy scale for adults [34] and the one for children 8–12 years [35] both consist of 29 items. Our comprehensive 47-items questionnaire is slightly longer than these scales, but fits within the range that Carroll et al. [31] have found. In addition, a completion time of less than 15 min (16–17 min including socio-economic background variables and other questions) seems reasonable.

### Strengths and limitations

A strength of the current study is the comprehensive and structured approach that was used to develop the questionnaire. The target group was involved in the development and participated in two studies with about 500 adolescents who were deliberately of different ages, school levels, and various schools for validating and optimizing the questionnaire. We acknowledge that our study would have benefitted from a larger and more diverse sample of adolescents participating in the development stage. A distinctive point of our questionnaire is that the measurement of knowledge is based on multiple-choice questions, coming closer to actual knowledge. Whereas food and nutrition knowledge is considered an important aspect of food literacy [23, 27–29, 37, 39, 41, 54], food literacy scales usually apply true-false answers or Likert scales for knowledge questions [26, 35, 37], which measures knowledge on a more general level.

A limitation of the study is that temporal stability was not assessed. Given the increased focus on the General Data Protection Regulation and the need to engage adolescents who are less interested in food and nutrition, it was decided to use an anonymous survey. Therefore, we were unable to link individual answers and performing a test-retest was not possible. Future studies using this questionnaire should include a test-retest to explore its temporal stability. We used convenience sampling in the validation studies, which resulted in a relatively higher educated sample in the first validation study and a relatively lower educated sample in the second study. Moreover, participating schools were mainly located in the northern and middle part of the Netherlands. Therefore, the results may not be completely representative of the Dutch adolescent population. For the gender differences analysis, 10% of the sample could not be included, since these participants answered ‘other’ or ‘don’t want to say’ to this question.

Another limitation is that our questionnaire was not validated against other outcomes, such as healthy eating or sustainable food choices. Food literacy is regarded as an essential requirement for supporting healthy and sustainable choices [19, 29, 55]. Previous food literacy scales have been cross-sectionally linked with nutritional outcomes [30, 34, 37] and higher food literacy in secondary school pupils has been linked to healthier dietary practices [32]. Yet, other studies have shown mixed findings or no association between food literacy and adolescents’ dietary intake [33]. Further research should investigate whether our food literacy scale is related to adolescents’ healthy and sustainable food choices.

Finally, the food literacy questionnaire makes use of self-report, which is prone to social desirability. Effort was made to minimize this as much as possible by using an anonymous survey, by using objective phrasing, and stimulating the adolescents to answer honestly by not being present as a researcher. Students may also over- or underestimate their own capacities. Since the sample sizes were relatively large, we expect that under- and overestimation levelled out on such a scale, with no or minor influence on the findings.

### Implications & future research

The broad applicability of the instrument (age, gender, educational level) and a completion time of less than 15 min implies that the questionnaire can be widely used in research and practice. First, it can be applied in cross-sectional studies to identify the most important aspects that nutrition programs should focus on (prioritization). Second, the tool can also be utilized in longitudinal studies to measure food literacy within a certain population or country over time, investigating time and national policy effects (trends). Finally, the food literacy questionnaire could be deployed in measurements before and after implementing healthy and sustainable eating interventions (effect measure). Its suitability for these applications should be further tested in future studies.

Additional research is needed to explore its use in other Western countries. It would also be interesting to explore its applicability in tertiary education, such as secondary vocational education or higher professional education. These education levels usually encompass a mixture of adolescents and young adults. Looking at the high overweight and obesity rates among young adults [3, 56], this group forms an important target for healthy and sustainable eating interventions. Finally, it would be valuable for future research to explore whether the sub-concepts are reliable and sensitive enough on their own. For example, it would be interesting to investigate whether the sub-concept *Preparation skills* (5 items) would show differences before and after implementing a cooking program for adolescents.

Finally, it is important to realize that food literacy is a key asset for adolescents to enable a switch towards healthier and more sustainable eating habits, but it is also crucial to change the environment to support healthy and sustainable food choices [19, 48, 49].

## Conclusions

The developed food literacy questionnaire for adolescents had satisfactory internal consistency and validity. It is a comprehensive questionnaire consisting of 47 items covering the concepts knowledge, skills, and the ability to deal with influences from the environment. It is suitable for different ages (12–18 years) and different school levels and takes about 15 min to complete. This broad applicability underpins the use of this questionnaire in large cross-sectional and longitudinal studies, as well as experimental studies evaluating the effectiveness of interventions. Future research should explore its application in other contexts.

## Supplementary Information

Supplementary Material 1.

## Data Availability

The datasets used and/or analysed during the current study are available from the corresponding author on reasonable request. The full questionnaire is available upon request.

## Abbreviations

COM-B: Capability, Opportunity, Motivation and Behaviour (model)
CA: Cronbach’s alpha
SD: Standard Deviation
N.A.: Not Applicable
SPSS: Statistical Package for the Social Sciences
