# Supplementary material for: Development and validation of a comprehensive food literacy questionnaire for adolescents integrating knowledge, skills, and environmental influences
Source: BMC Public Health. 2026 Mar 13;26:1294. doi: 10.1186/s12889-026-26969-2 (PMC13097919; doi:10.1186/s12889-026-26969-2)
Supplement: Supplementary file 1 — Supplementary Material 1. [file 12889_2026_26969_MOESM1_ESM.docx]

## Appendix 1. Results Dutch Food Literacy Questionnaire for Adolescents (12-18 years)

| **nr.** | **KNOWLEDGE** | **N** | **% correct** |
| --- | --- | --- | --- |
|  | **Knowledge on link between nutrition and health** |  |  |
| *Q1* | *What happens to excess food in your body if you eat more than you need?* | *658* | *88%* |
| *Q2* | *What disease could you get from this?* | *658* | *79%* |
|  | **Knowledge about healthy eating** |  |  |
| *Q3* | How much vegetables should someone of your age eat at least daily to stay healthy? | 652 | 68% |
| *Q4* | How much fruit should someone of your age eat daily to stay healthy? | 652 | 70% |
| *Q5* | Which product groups are included in the Dutch Wheel of Five? | 644 | 65% |
| *Q6* | Which nutrients provide energy? | 644 | 46% |
| *Q7* | *Why is it important to eat foods from different sections of the Dutch Wheel of Five every day?* | *631* | *94%* |
| *Q8a* | *Water ice vs. ice cream* | *631* | *87%* |
| *Q8b* | *Chips vs. unsalted nuts* | *631* | *96%* |
| *Q8c* | *Apple vs. fruit smoothie* | *631* | *87%* |
| *Q8d* | *Filled cookie vs. speculaas (spiced cookie)* | *631* | *75%* |
| *Q8e* | *Chicken breast vs. smoked sausage* | *631* | *89%* |
| *Q9* | Which foods are high in protein? | 626 | 50% |
| *Q10* | *Which product contains the most vitamin C?* | *626* | *6%* |
| *Q11* | Why are vitamins important? | 620 | 84% |
| *Q12* | *Why is it better to choose brown rice instead of white rice?* | *620* | *88%* |
| *Q13* | What do you need when you do sports daily ? | 620 | 66% |
|  | **Knowledge about foods** |  |  |
| *Q14* | *What’s true about canned vegetables?* | *610* | *85%* |
| *Q15* | *What’s the most sustainable choice?* | *610* | *85%* |
| *Q16* | What type of meat is best to choose if you want to consider the environment? | 608 | 43% |
| *Q17* | What’s a good substitute for meat in a meal? | 608 | 67% |
| *Q18* | *To which product is salt added?* | *601* | *89%* |
| *Q19* | What do the expiry dates "Best Before" and "Use-By" mean? | 601 | 78% |
| *Q20* | The packaging of a product lists its ingredients. What’s true about this? | 603 | 63% |
| *Q21* | What is best to cook briefly in little water to maintain the nutrients? | 603 | 56% |
| *Q22* | *What’s true about halal meat?* | *602* | *86%* |
| Q23 | What does the Nutri-Score indicate? | 602 | 46% |
| Q24 | What do these logos mean (Fairtrade, organic)? | 590 | 40% |

|  | **Items** | **N** | **Mean** | **SD** | **CA** |
| --- | --- | --- | --- | --- | --- |
|  | **SKILLS** |  | |  |  |
|  | **Planning skills** |  | |  | **0.770** |
| Q25 | I can tell whether what I eat and drink matches what I should be eating and drinking. | 588 | 3.87 | 0.98 |  |
| Q26 | I can explain why eating too much or too little isn’t good for my body. | 588 | 4.21 | 0.93 |  |
| Q27 | I can explain what someone should eat and drink to take care of the environment. | 588 | 3.46 | 1.13 |  |
| Q28 | I can explain the difference between organic and non-organic products. | 588 | 3.71 | 1.14 |  |
| Q29 | I can explain why some products come from other countries. | 588 | 4.13 | 0.94 |  |
| Q30 | I know how to consider the environment when choosing products in the store. | 654 | 3.39 | 1.16 |  |
| Q31 | I know where to find reliable information about products. | 654 | 3.34 | 1.17 |  |
|  | **Selection skills** |  |  |  | **0.755** |
| Q32 | When I am buying food myself, I know how to make a healthy choice. | 591 | 3.91 | 0.97 |  |
| Q33 | I can think of what products I need for an evening meal before I go to the supermarket. | 591 | 4.10 | 0.95 |  |
| Q34 | I can explain why making a shopping list is helpful. | 591 | 4.20 | 1.02 |  |
| Q35 | I can determine how much products to buy to prepare a meal with. | 591 | 3.85 | 0.96 |  |
| Q36 | I can buy products with little money to make a healthy dinner. | 591 | 3.76 | 1.05 |  |
| *Q37* | *I know where on the packaging it says what ingredients are in the product.* | *654* | *4.48* | *0.86* |  |
|  | **Preparation skills** |  |  |  | **0.829** |
| Q38 | I can tell from a recipe if it’s a healthy dinner. | 579 | 4.23 | 0.90 |  |
| Q39 | I can cut an onion. | 579 | 4.41 | 1.04 |  |
| Q40 | *I can cut an apple.* | *579* | *4.51* | *0.97* |  |
| Q41 | *I know how to use a mixer.* | *579* | *4.64* | *0.77* |  |
| Q42 | *I can weigh 100 grams of rice.* | *579* | *4.56* | *0.84* |  |
| Q43 | I can cook a simple dinner. | 579 | 4.37 | 0.95 |  |
| Q44 | I can make a vegetarian dinner (without meat). | 579 | 3.84 | 1.37 |  |
| Q45 | *I can explain why you should wash your hands before cooking.* | *579* | *4.62* | *0.77* |  |
| Q46 | I can explain why you should cut vegetables and meat on separate cutting boards. | 579 | 4.22 | 1.14 |  |
|  | **Storage skills** |  |  |  | **0.748** |
| Q47 | I can store food in the right place so it does not spoil. | 574 | 4.28 | 0.89 |  |
| Q48 | I can see, smell or feel whether fresh meat, fish, vegetables or fruit are still good to eat. | 574 | 3.88 | 1.09 |  |
| Q49 | I can explain why food spoils. | 574 | 3.97 | 1.06 |  |
| Q50 | I can explain how food waste affects the environment. | 574 | 3.82 | 1.11 |  |
| *Q51* | *I know how I can prevent food waste.* | *654* | *3.94* | *0.98* |  |
|  | **SOCIAL ENVIRONMENT** |  |  |  | **0.654** |
|  | **Social influences** | | | | **0.551** |
| Q52 | At a party, I can resist food if I don’t feel like eating. ^(SC)^ | 619 | 4.25 | 1.03 |  |
| Q53 | When my friends are buying snacks, I can join them without buying anything myself. ^(SC)^ | 619 | 3.71 | 1.30 |  |
| Q54 | I can explain why eating at the table is better than eating while watching TV or gaming. ^(HSCI)^ | 619 | 3.31 | 1.44 |  |
| Q55 | I can give examples of how influencers promote unhealthy products. ^(HSCI)^ | 619 | 3.64 | 1.13 |  |
| Q56 | I can take dietary restrictions of another person into account. ^(SC)^ | 619 | 4.09 | 1.00 |  |
|  | **Balance** |  | | | **0.443** |
| Q57 | I can feel when I’m full and I’ve eaten enough. ^(SC)^ | 616 | 4.13 | 0.91 |  |
| Q58 | I'm open to taste foods that I have never tasted before. ^(HSCI)^ | 616 | 3.83 | 1.00 |  |
| Q59 | When I’ve eaten a lot, I can manage to eat a little less afterwards. ^(SC)^ | 616 | 3.74 | 1.01 |  |
|  | **PHYSICAL ENVIRONMENT** |  | | | **0.581** |
| Q60 | I can resist temptation when I smell delicious food on the street (like from a snack bar or bakery). ^(SC)^ | 612 | 3.86 | 1.00 |  |
| Q61 | When I see an ad about a product, I can determine whether it's healthy or less healthy. ^(HSCI)^ | 612 | 3.88 | 0.88 |  |
| Q62 | I can cite examples of dishes from different cultures. ^(HSCI)^ | 612 | 4.05 | 0.96 |  |
| Q63 | I can explain what terms like halal and kosher mean. ^(HSCI)^ | 612 | 3.31 | 1.29 |  |

Items presented in *italics* were excluded in study 2 and the final version of the questionnaire;

Based on results study 1 recoded to Self-control ^(SC)^ and Handling socio-cultural influences ^(HSCI)^;

The questions in Qualtrics were differently ordered for optimal logic and variation reasons. In study 1: Q1, Q2, Q30, Q31, Q37, Q51, Q3-Q13, Q52-Q63, Q14-Q24, Q32-Q36, Q25-Q29, Q38-Q50.
